# Enhanced DNA barcode datasets for *Aristida* grasses: new whole genome skimming data for the protected species *Aristida triseta* Keng integrated with public sequences

**DOI:** 10.1016/j.dib.2026.113043

**Published:** 2026-06-28

**Authors:** Xiaoman Wang, Kai Mu, Chao Xu, Zhengge Zhu, Xueying Yang, Jin Zhang

**Affiliations:** a College of Life Sciences, Hebei Normal University, Shijiazhuang, 050024, China; b Engineering Research Centre of Forestry Biotechnology of Jilin Province, College of Forestry, Beihua University, Jilin, 132013, China; c Key Laboratory of Systematic and Evolutionary Botany/ State Key Laboratory of Plant Diversity and Specialty Crops, Institute of Botany, Chinese Academy of Sciences, Beijing, 100093, China; d China National Botanical Garden, Beijing, 100093, China; e National Engineering Laboratory for Forensic Science, Key Laboratory of Forensic Genetics, Institute of Forensic Science, Ministry of Public Security, Beijing, 100038, China

**Keywords:** *Aristida triseta*, DNA barcode, Chloroplast genome, Internal transcribed spacer (ITS), Phylogeny

## Abstract

*Aristida triseta* Keng is a Class II national protected wild plant in China, possessing significant ecological and economic importance. In this study, the complete chloroplast genome of *A. triseta* was sequenced using the DNBSEQ-T7 platform. The chloroplast genome is a circular molecule with a total length of 138,428 bp, displaying the typical quadripartite structure found in Poaceae. It consists of a large single-copy (LSC) region of 80,413 bp, a small single-copy (SSC) region of 12,517 bp, and a pair of inverted repeat regions (IRa and IRb) of 22,749 bp each. A total of 133 genes were annotated, comprising 87 protein-coding genes (PCGs), 38 transfer RNA (tRNA) genes, and 8 ribosomal RNA (rRNA) genes. Meanwhile, we screened and acquired sequence data for six core DNA barcode regions (*rbcL, matK, ndhF, rpl16, trnL-trnF*, and ITS) from the sequencing data. By integrating these sequences with publicly available *Aristida* sequences from public databases, we constructed six enhanced DNA barcode datasets for the genus *Aristida*. Similarity analysis using blastn and phylogenetic analyses showed that each barcode sequence of *A. triseta* efficiently matched homologous sequences from the genus *Aristida*, exhibiting heterogeneous phylogenetic signals. The six enhanced DNA barcode datasets enrich the genetic resources of *Aristida*, providing a foundation for molecular breeding, genetic diversity analysis, and phylogenetic research of *A. triseta*, while also serving as a useful resource for developing molecular markers for species discrimination and taxonomic studies within the genus *Aristida*.

Specifications Table

**Table:** 

Subject	Biology
Specific subject area	Omics: Genomics
Type of data	Tables, Figures. Raw, Analyzed.
Data collection	Total genomic DNA was extracted from dried leaf material of *Aristida triseta* Keng (voucher PE-00260509, PE herbarium) using a modified CTAB method. Whole genome skimming sequencing was performed on the DNBSEQ-T7 platform. Raw reads were processed with fastp v0.23.1. The chloroplast genome was de novo assembled by GetOrganelle v1.7.7.1, annotated with Plann v1.1, and visualized by CPGView v1.0. Six DNA barcode regions (*rbcL, matK, ndhF, rpl16, trnL-trnF*, ITS) were extracted, integrated with GenBank *Aristida* sequences, aligned by MAFFT v7.505, and manually corrected to form six enhanced datasets. Local blastn similarity analysis was conducted, followed by phylogenetic analysis using IQ-TREE 2.3.6.
Data source location	City: Yushu County, Qinghai Province. Country: China. The voucher specimen was deposited at the herbarium of Institute of Botany, Chinese Academy of Sciences (PE) under voucher number PE-00260509.
Data accessibility	Repository name: NCBI (National Center for Biotechnology Information). Direct URL to data: https://www.ncbi.nlm.nih.gov/nucleotide/PZ161599 https://www.ncbi.nlm.nih.gov/biosample/SAMN56514143 https://www.ncbi.nlm.nih.gov/bioproject/PRJNA1437527 https://www.ncbi.nlm.nih.gov/sra/?term=SRR37669847 The Science Data Bank links to the six enhanced DNA barcode datasets can be accessed at: https://doi.org/10.57760/sciencedb.31238">https://doi.org/10.57760/sciencedb.31238
Related research article	None.

## 1 Value of the Data

• This study provides the complete chloroplast genome sequence of *Aristida triseta*, filling a critical gap in genomic resources for the genus *Aristida*. The data offer robust molecular evidence to support species identification and phylogenetic inference involving *Aristida* species.
• The six enhanced DNA barcode datasets (*rbcL, matK, ndhF, rpl16, trnL*-*trnF*, and ITS) enrich the molecular marker resources available for *Aristida* grasses, serving as valuable tools for plant molecular identification and systematic studies.
• These integrated datasets can be applied to genetic diversity analyses within *Aristida* and contribute to conservation genetics research on the protected species *A. triseta*, thereby supporting the development of evidence-based conservation strategies.

## 2 Background

**Fig. 1:**
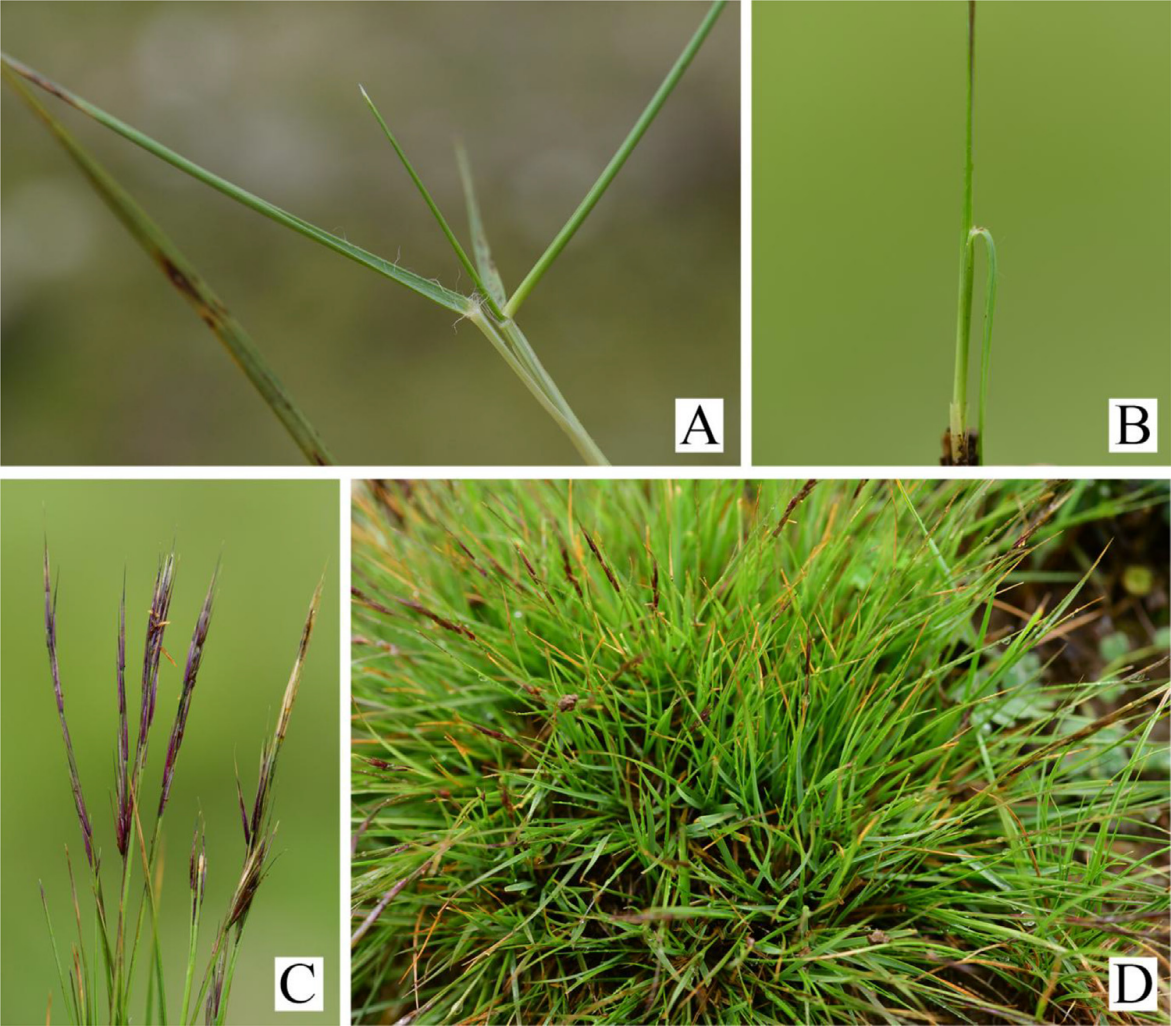
Morphology and habitat of *Aristida triseta* Keng. A. Leaf (photo by Zi Wang); B. Basal stem; C. Spikelets; D. Tufted habit (photos B-D by Xinxin Zhu).

*Aristida triseta* Keng belongs to the genus *Aristida* in the family Poaceae (Fig. 1). It is an endemic and protected species in western China, mainly distributed in Qinghai, Tibet, and Sichuan. As a Class II national protected wild plant in China, this species plays an important role in grassland restoration and serves as a valuable forage resource, thus possessing high ecological and economic significance.

Chloroplast genomes are generally maternally inherited and characterized by a highly conserved quadripartite circular structure. Although the overall sequence conservation of chloroplast genomes is relatively high, they contain abundant variable sites. Consequently, the highly divergent regions of the chloroplast genome have been extensively applied in phylogenetic reconstruction and DNA barcoding research [1]. Early phylogenetic analyses of the subfamily Aristidoideae (comprising three monophyletic genera: *Aristida, Stipagrostis*, and *Sartidia*) based on chloroplast genes (*trnL-F, rpl16*) and the nuclear gene ITS have clarified that *Aristida* is sister to the clade formed by *Stipagrostis* and *Sartidia* [2,3]. Subsequent studies based on complete chloroplast genomes have further validated this relationship.

Although several taxonomic and phylogenetic studies have been conducted on the genus *Aristida*, gaps remain in the chloroplast genome and DNA barcode data of *A. triseta* based on current public databases (NCBI GenBank) and available literature. In this study, we generated whole genome skimming data of *A. triseta*, assembled its complete chloroplast genome, and combined six DNA barcode regions (*rbcL, matK, ndhF, rpl16, trnL-trnF*, and ITS). These sequences were integrated with publicly available *Aristida* sequences to construct six enhanced DNA barcode datasets. This study provides valuable genetic resources for molecular marker development, phylogenetic reconstruction of the genus *Aristida*, and conservation genetic research on *A. triseta*.

## 3 Data Description

Whole genome skimming generated approximately 67.06 GB of raw sequencing data, consisting of 447,036,758 reads with an average GC content of 46.9%. The sequencing quality was generally high, with Q20 and Q30 values reaching 99.25% and 98.13%, respectively.

**Fig. 2:**
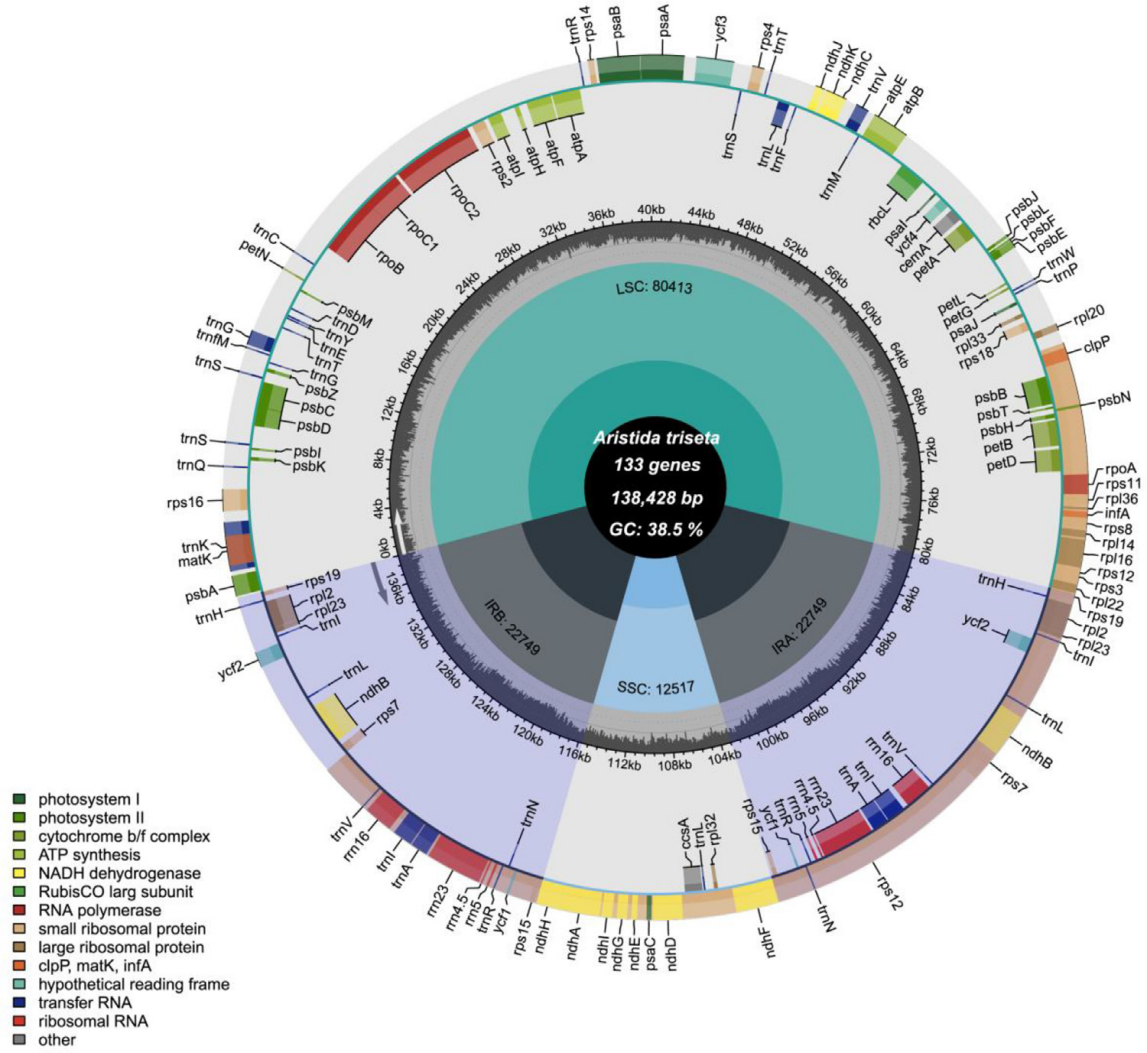
Genetic map of the complete chloroplast genome of *Aristida triseta*. The central region indicates the total number of annotated genes (133), genome length (138,428 bp), and GC content (38.5%). Moving outward, the first circle depicts the large single-copy (LSC), small single-copy (SSC), and inverted repeat (IRa and IRb) regions, with darker regions corresponding to GC content. The second circle represents the GC content profile. Genes outside the third circle are transcribed counterclockwise, and those inside are transcribed clockwise. Genes are color-coded by functional category, with the legend shown in the bottom left.

The complete chloroplast genome of *Aristida triseta* Keng is a circular molecule with a length of 138,428 bp and a GC content of 38.5%. It exhibits a typical quadripartite structure, containing a large single-copy (LSC) region of 80,413 bp, a small single-copy (SSC) region of 12,517 bp, and a pair of inverted repeat (IR) regions (IRa and IRb) of 22,749 bp each (Fig. 2). A total of 133 genes were annotated in the chloroplast genome of *A. triseta*, including 87 protein-coding genes (PCGs), 38 transfer RNA (tRNA) genes, and 8 ribosomal RNA (rRNA) genes (Table 1). Among these annotated genes, 18 genes (including *ndhA, ndhB, petB, petD, atpF, rpl2, rpl16, rps16, trnA-UGC, trnG-UCC, trnI-GAU, trnK-UUU, trnL-UAA*, and *trnV-UAC*) possess a single intron, while two genes (*rps12* and *ycf3*) contain two introns each. It has been reported that the degree of degradation of certain genes (e.g., *accD, ycf1, ycf2*) varies among species of the subfamily Aristidoideae [4], and the *accD* gene was not annotated in this chloroplast genome.

**Table 1:** Classification of the *Aristida triseta* genes after annotation of the chloroplast genome. The annotated genes were categorized according to their function.

Category	Gene group	Gene name
Photosynthesis	Subunits of photosystem I	*psaA, psaB, psaC, psaI, psaJ*
	Subunits of photosystem II	*psbA, psbB, psbC, psbD, psbE, psbF, psbH, psbI, psbJ, psbK, psbL, psbM, psbN, psbT, psbZ*
	Subunits of NADH dehydrogenase	ndhA*, ndhB* (2), *ndhC, ndhD, ndhE, ndhF, ndhG, ndhH, ndhI, ndhJ, ndhK*
	Subunits of cytochrome b/f complex	petA, petB*, petD*, petG, petL, petN
	Subunits of ATP synthase	atpA, atpB, atpE, atpF*, atpH, atpI
	Large subunit of rubisco	*rbcL*
Self-replication	Proteins of large ribosomal subunit	rpl2* (2), rpl14, rpl16*, rpl20, rpl22, rpl23 (2), *rpl32, rpl33, rpl36*
	Proteins of small ribosomal subunit	*rps2, rps3, rps4, rps7* (2), *rps8, rps11, rps12*⁎⁎ (2), *rps14, rps15* (2), rps16*, rps18, rps19 (2)
	Subunits of RNA polymerase	*rpoA, rpoB, rpoC1, rpoC2*
	Ribosomal RNAs	*rrn4.5* (2), *rrn5* (2), *rrn16* (2), *rrn23* (2)
	Transfer RNAs	trnA-UGC* (2), trnC-GCA, trnD-GUC, trnE-UUC, trnF-GAA,trnG-GCC, trnG-UCC*, trnH-GUG (2), *trnI-CAU* (2), trnI-GAU* (2), trnK-UUU*, trnL-CAA (2), trnL-UAA*, *trnL-UAG, trnM-CAU, trnN-GUU* (2), *trnP-UGG, trnQ-UUG, trnR-ACG* (2), *trnR-UCU, trnS-GCU, trnS-GGA, trnS-UGA, trnT-GGU, trnT-UGU, trnV-GAC* (2), trnV-UAC*, trnW-CCA, trnY-GUA,trnfM-CAU
Other genes	Maturase	*matK*
	Protease	*clpP*
	Envelope membrane protein	*cemA*
	c-type cytochrome synthesis gene	*ccsA*
	Translation initiation factor	*infA*
Genes of unknown function	Conserved hypothetical chloroplast ORF	#*ycf1* (2),#*ycf2* (2), *ycf3*⁎⁎*, ycf4*

Notes: Gene

⁎ Gene with one intron; Gene

⁎⁎ Gene with two introns; #Gene: Pseudo gene; Gene (2): multi-copy genes (copy number).

Six DNA barcode sequences, including the *rbcL, matK, ndhF, rpl16*, and *trnL-trnF* regions from the assembled chloroplast genome of *A. triseta*, as well as the nuclear ITS region, were obtained. These sequences were combined with related sequences of the genus *Aristida* retrieved from public databases to construct six enhanced DNA barcode datasets for local sequence alignment and phylogenetic inference. The total number of sequences and species (excluding outgroups) in the final datasets were as follows: ITS (193, 98), *matK* (127, 73), *rbcL* (149, 74), *rpl16* (192, 102), *ndhF* (84, 52), and *trnL-trnF* (198, 99). *Sartidia perrieri, S. dewinteri*, and *S. isaloensis* were used as the outgroup for the five chloroplast markers. The outgroup for the nuclear ITS region included *S. jucunda, S. perrieri, S. dewinteri*, and *S. isaloensis*. The six aligned DNA barcode datasets are available at https://doi.org/10.57760/sciencedb.31238.

Using the six DNA barcode sequences of *A. triseta* generated in this study as queries, a local nucleotide database was constructed based on the enhanced DNA barcode datasets, and sequence similarity analysis was performed using the blastn program. The results showed that the percent identity and bitscores of each DNA barcode region were as follows: the ITS region had a percent identity ranging from 96.17% to 98.99% and bitscores of 974–1,062; the *matK* region had a percent identity ranging from 96.04% to 99.87% with bitscores of 1,352–2,820; the *rbcL* region ranged from 98% to 99.87% in percent identity and 649–2,483 in bitscores; the *ndhF* region exhibited a percent identity of 95.37%–99.66% and bitscores of 412–3,819; the *rpl16* region had a percent identity of 96.88%–99.40% and bitscores of 1650–1,801; the *trnL-trnF* region showed a percent identity of 97.84%–100% with bitscores of 126–1,777. Notably, due to the varying lengths of relevant sequences in the reference database for each region, the start and end positions of the aligned sequences were inconsistent, resulting in differences in matched lengths. Consequently, some sequences exhibited high similarity but relatively low bitscores attributed to the short matched lengths.

**Fig. 3:**
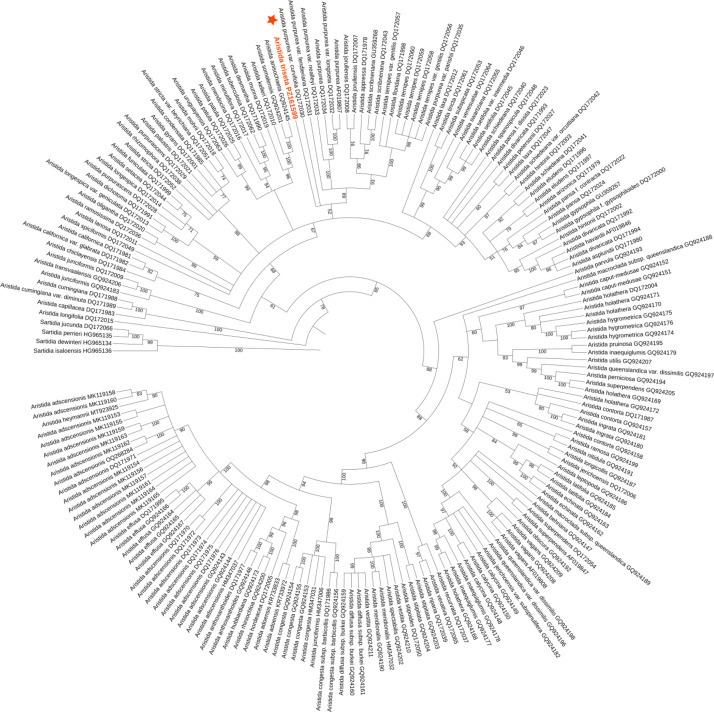
Maximum-likelihood (ML) phylogenetic tree inferred from ITS sequences of *Aristida triseta* and related species within the genus *Aristida*, with species of the genus *Sartidia* serving as the outgroup. Bootstrap support (BS) values (≥50%) are shown above the nodes.

**Fig. 4:**
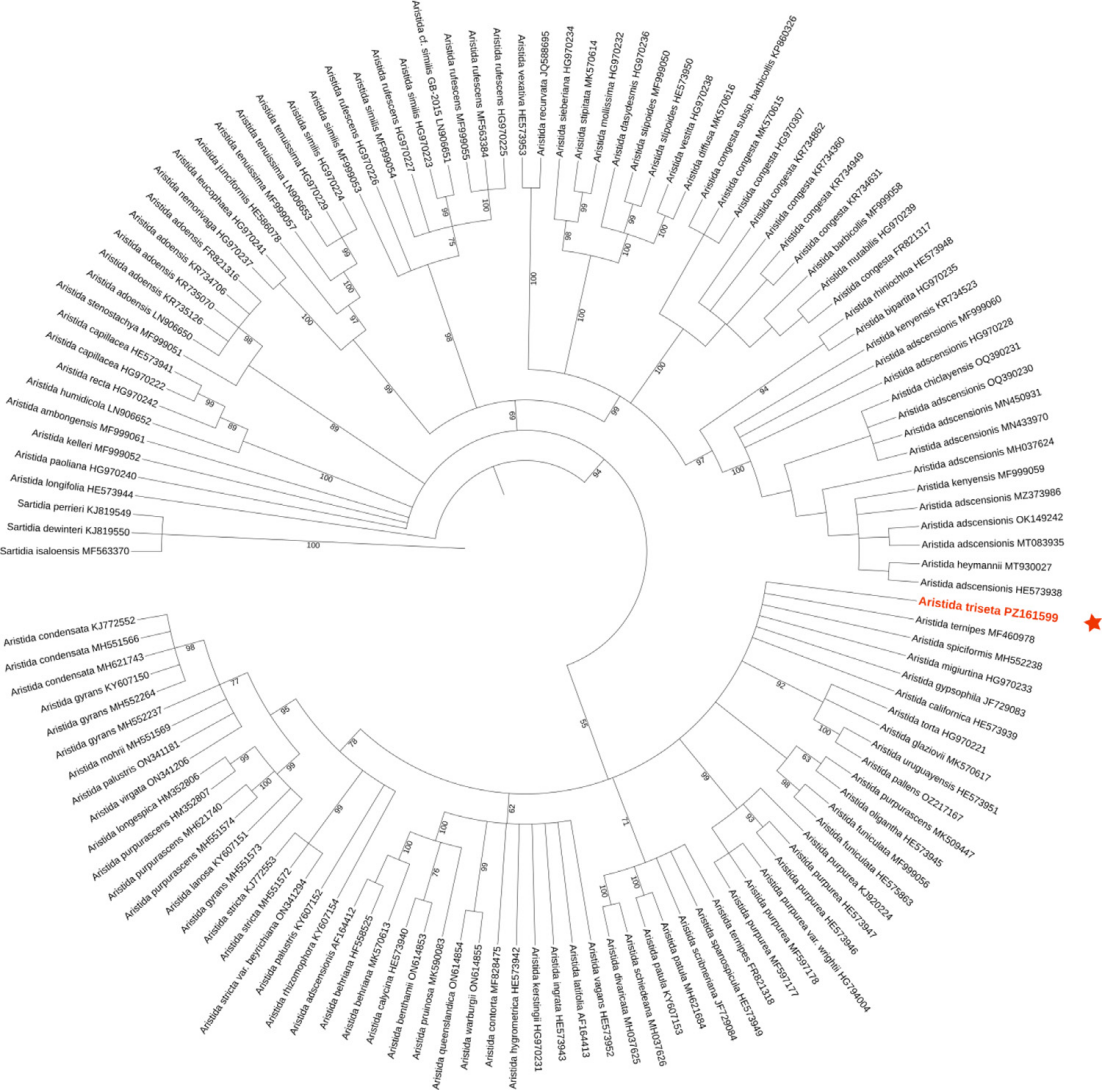
Maximum-likelihood (ML) phylogenetic tree inferred from *matK* sequences of *Aristida triseta* and related species within the genus *Aristida*, with species of the genus *Sartidia* serving as the outgroup. Bootstrap support (BS) values (≥50%) are shown above the nodes.

**Fig. 5:**
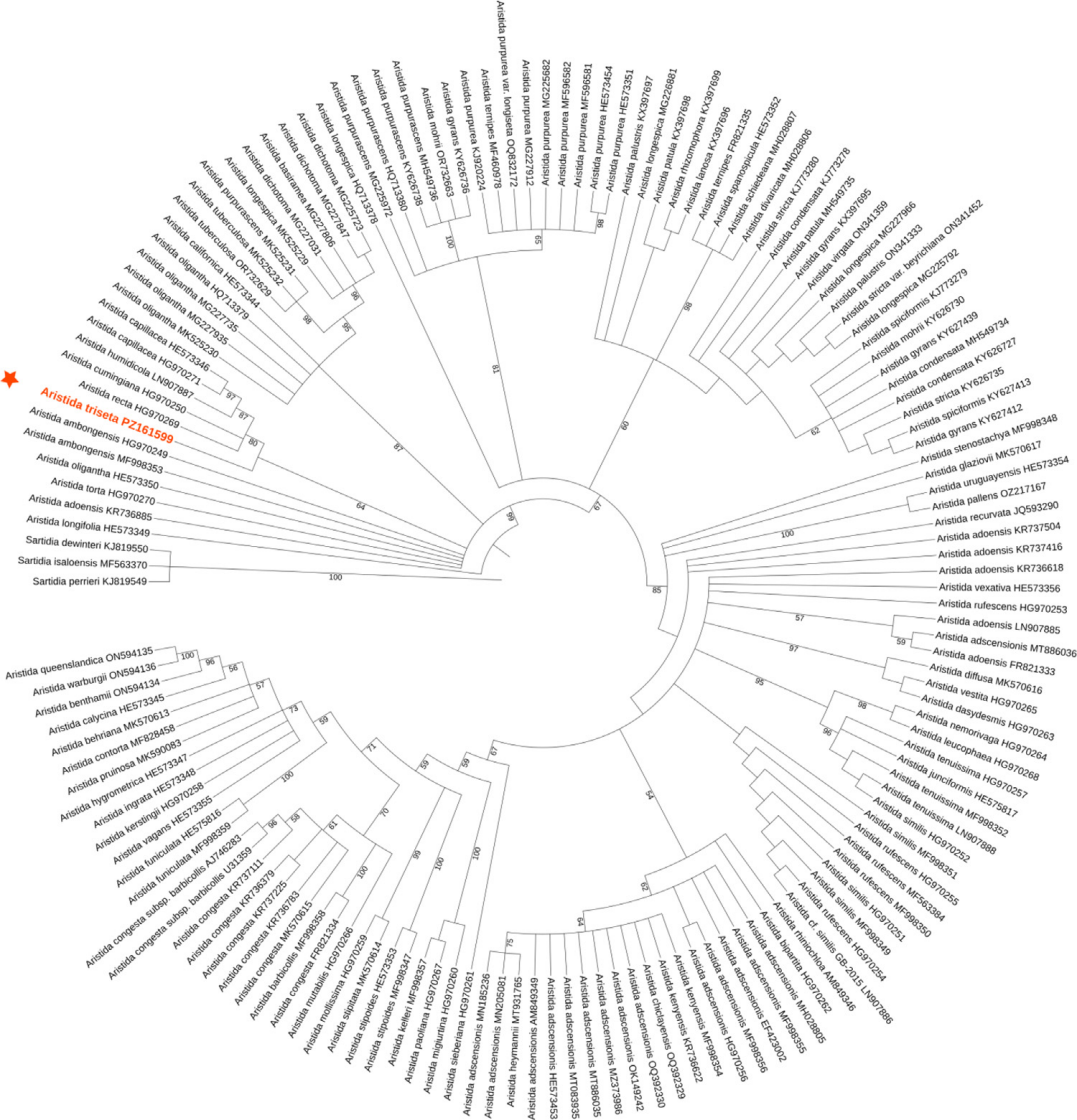
Maximum-likelihood (ML) phylogenetic tree inferred from *rbcL* sequences of *Aristida triseta* and related species within the genus *Aristida*, with species of the genus *Sartidia* serving as the outgroup. Bootstrap support (BS) values (≥50%) are shown above the nodes.

**Fig. 6:**
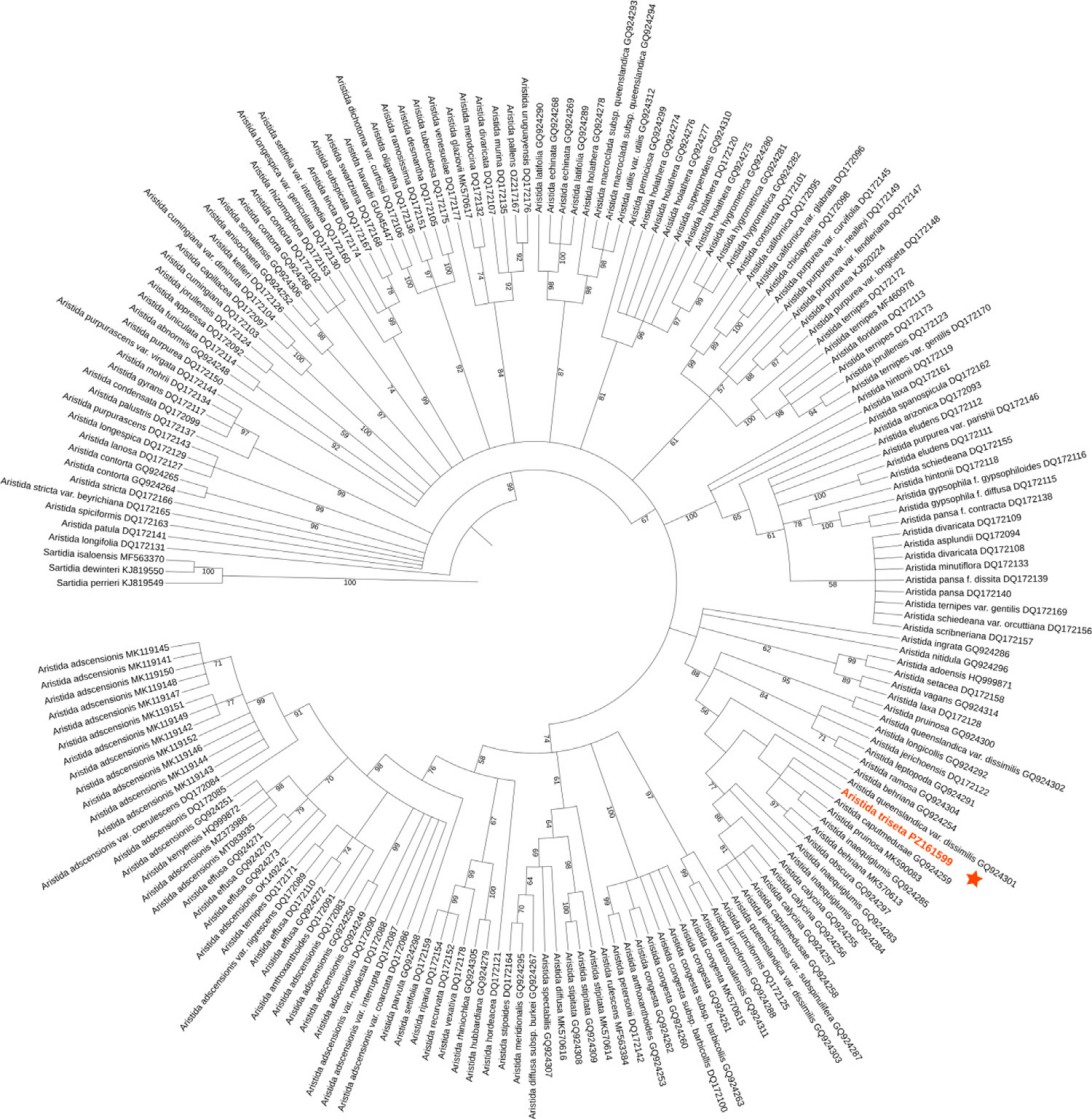
Maximum-likelihood (ML) phylogenetic tree inferred from *rpl16* sequences of *Aristida triseta* and related species within the genus *Aristida*, with species of the genus *Sartidia* serving as the outgroup. Bootstrap support (BS) values (≥50%) are shown above the nodes.

**Fig. 7:**
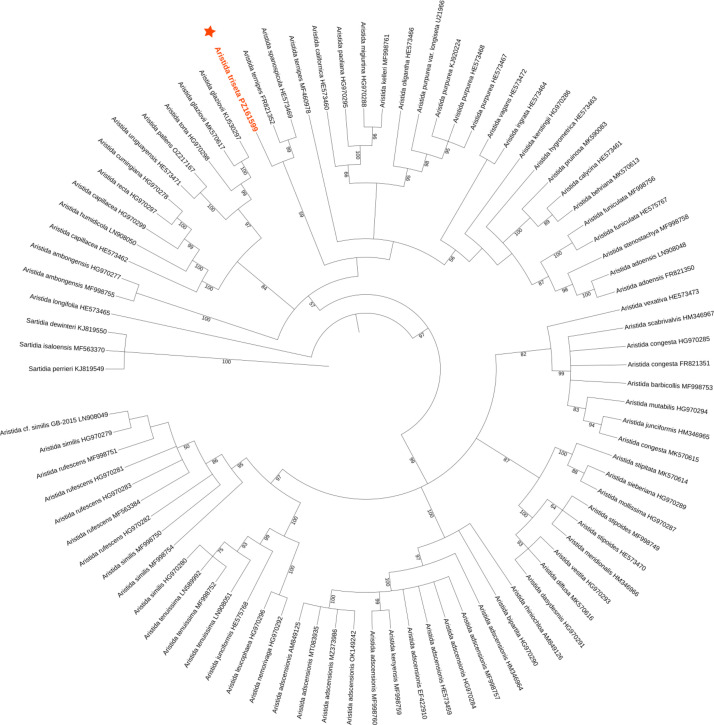
Maximum-likelihood (ML) phylogenetic tree inferred from *ndhF* sequences of *Aristida triseta* and related species within the genus *Aristida*, with species of the genus *Sartidia* serving as the outgroup. Bootstrap support (BS) values (≥50%) are shown above the nodes.

**Fig. 8:**
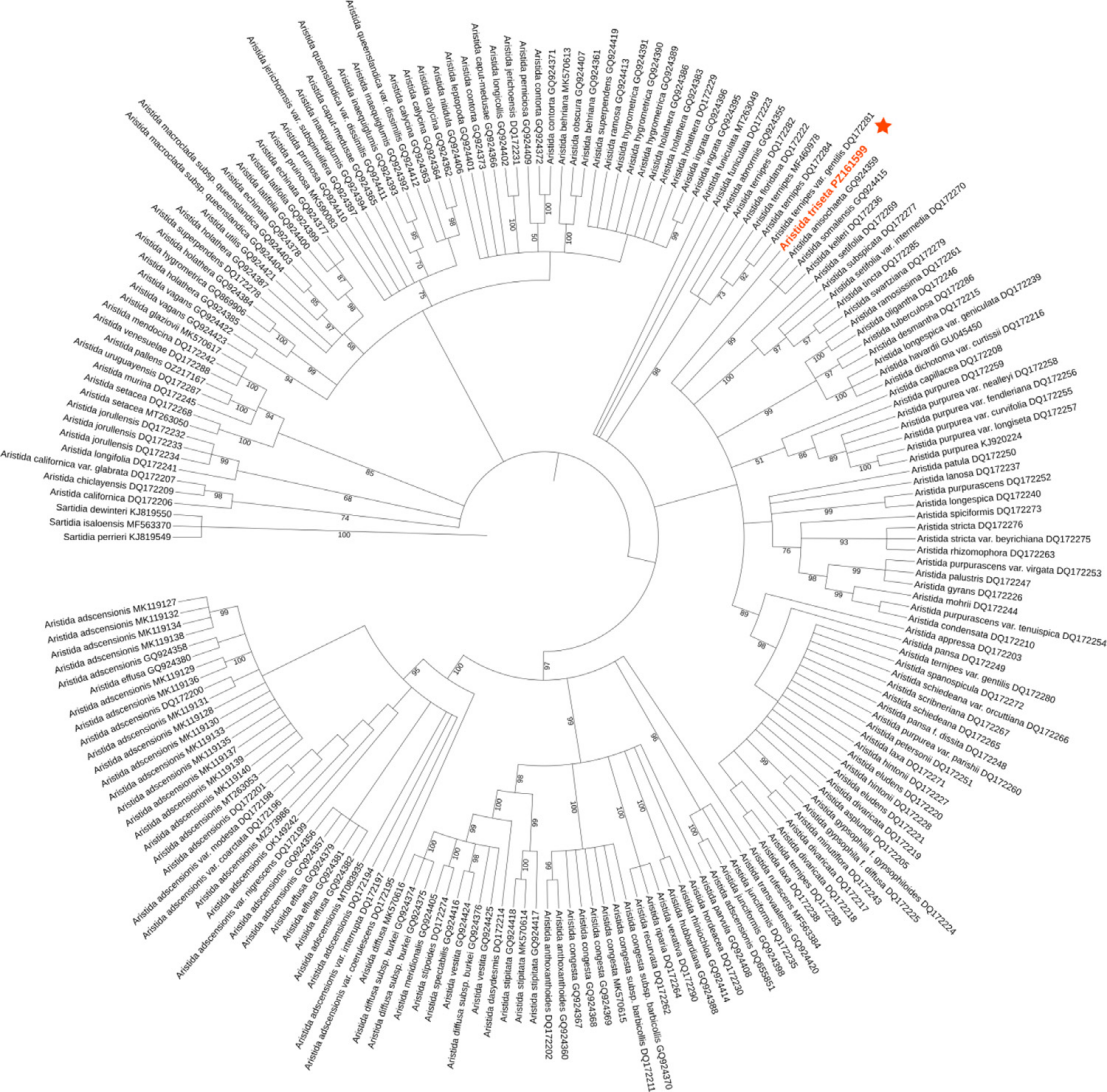
Maximum-likelihood (ML) phylogenetic tree inferred from *trnL-trnF* sequences of *Aristida triseta* and related species within the genus *Aristida*, with species of the genus *Sartidia* serving as the outgroup. Bootstrap support (BS) values (≥50%) are shown above the nodes.

Phylogenetic trees were constructed for all datasets using the maximum likelihood (ML) method, and the resulting topologies were shown in Fig. 3, Fig. 4, Fig. 5, Fig. 6, Fig. 7, Fig. 8. Phylogenetic analyses based on different DNA barcode sequences consistently placed *Aristida longifolia* at the base of the genus *Aristida*, which is consistent with previous reports [5]; however, the phylogenetic position of *A. triseta* varied among the trees. In the ITS tree, *A. triseta* clustered with the *A. purpurea* group [6] with 52% bootstrap support (Fig. 3). The *matK* tree grouped *A. triseta* together with several congeners, including *A. ternipe*s MF460978, *A. spiciformis* MH552238, *A. migiurtina* HG970233 and others, forming a clade with 55% support (Fig. 4). In the *rbcL* tree, *A. triseta* formed an independent branch sister to a clade comprising *A. recta* HG970269, *A. cumingiana* HG970250, *A. humidicola* LN907887, *A. capillacea* HG970271, and *A. capillacea* HE573346, with 64% support (Fig. 5). The *rpl16* tree placed *A. triseta* in a weakly supported clade (BS < 50%) together with *A. caputmedusae* GQ924259, *A. pruinosa* MK590083, *A. inaequiglumis* GQ924285, *A. behriana* MK570613, and *A. obscura* GQ924297 (Fig. 6). In the *ndhF* tree, *A. triseta* formed an independent branch which was resolved as sister to *A. ternipes* FR821352 and *A. spanospicula* HE573469, with 59% support (Fig. 7). The *trnL-trnF* tree grouped *A. triseta* with several congeners, including *A. anisochaeta* GQ924359, *A. somalensis* GQ924415, *A. kelleri* DQ172236 and others, within a poorly supported clade (BS < 50%) (Fig. 8). Overall, the six barcode regions exhibited heterogeneous phylogenetic signals, which may be partly attributed to data fragmentation in public databases.

## 4 Experimental Design, Materials and Methods

### 4.1 Plant materials and DNA extraction

*Aristida triseta* Keng was collected from Yushu County, Qinghai Province, China, in 1964, and the voucher specimen was deposited at the Herbarium of the Institute of Botany, Chinese Academy of Sciences (PE) under accession number PE-00260509. Total genomic DNA was extracted from leaf tissue using the modified CTAB (mCTAB) method [7]. Specifically, we extended the lysis incubation time to 90 min at 65°C, increased the sample amount, and reduced the number of washing steps with 75% ethanol. DNA quality was examined by 1% agarose gel electrophoresis, and DNA concentration was determined using a NanoDrop spectrophotometer at 260 nm.

### 4.2 Sequencing and sequence analyses

Whole genome skimming sequencing was carried out on the DNBSEQ-T7 platform, yielding 150 bp paired-end reads. Raw sequencing reads were quality-filtered using fastp v0.23.1 [8] to remove low-quality bases and adapters, retaining reads with Phred quality score ≥ 20 and length ≥ 50 bp. Automatic paired-end adapter detection, 5′ and 3′ terminal low-quality trimming, and paired-read error correction were performed during filtering. The filtered reads were assembled into a circular chloroplast genome and the internal transcribed spacer (ITS) region using GetOrganelle v1.7.7.1 [9]. Chloroplast sequences were assembled with the seed database specific for embryophyte plastid genomes and 10 iterative recruitment cycles; nuclear ribosomal ITS sequences were separately recovered relying on the seed dataset of embryophyte nuclear rRNA.

To adjust the quadripartite structure of the newly assembled chloroplast genome, the sequence was aligned with all available chloroplast genomes of the genus *Aristida* retrieved from the National Center for Biotechnology Information (NCBI) GenBank database using MEGA v12 [10]. The chloroplast genome of *Aristida* adscensionis (NCBI accession number: MZ373986) was selected as the reference for annotation, which was performed using Plann v1.1 [11] to predict protein-coding genes (PCGs), transfer RNA (tRNA) genes, and ribosomal RNA (rRNA) genes. A circular map of the *A. triseta* chloroplast genome was constructed with CPGView v1.0 [12].

Sequences of five chloroplast regions (*rbcL, matK, ndhF, rpl16*, and *trnL-trnF*) were extracted from the annotated chloroplast genome. The ribosomal internal transcribed spacer (ITS), comprising ITS1, 5.8S rDNA, and ITS2, was identified and extracted from ribosomal DNA (rDNA) sequences using ITSx [13]. These six regions were selected as core DNA barcodes for *Aristida* in this study, as they are the most abundantly represented in GenBank and cover the largest number of *Aristida* species sequenced to date. The complete chloroplast genome sequence of *Aristida triseta* has been submitted to GenBank under accession number PZ161599. The raw sequencing reads (fastq files) have been deposited in the NCBI Sequence Read Archive (SRA) under BioProject accession number PRJNA1437527, SRA accession number SRR37669847, and BioSample accession number SAMN56514143.

### 4.3 Sequence similarity search and phylogenetic analysis

The six DNA sequences of *A. triseta* were combined with publicly available *Aristida* sequences downloaded from GenBank. Multiple sequence alignment and curation were performed using MAFFT v7.505 [14] with automatic algorithm selection to construct enhanced DNA barcode datasets for *Aristida*.

For each of the six barcode regions, a nucleotide BLAST database was constructed using the makeblastdb command from the BLAST+ suite [15]. Sequence similarity searches were conducted using the blastn command, hits were filtered at an E-value threshold of 1e-10, and the top 100 best matches were retained for each query. The output fields included query sequence ID, subject sequence ID, query start and end positions, subject start and end positions, percent identity, and bitscore. Results were sorted in descending order by percent identity and are presented in Supplementary Tables S1-S6.

Maximum likelihood (ML) phylogenetic trees were constructed for the six datasets using IQ-TREE 2.3.6 [16]. The optimal nucleotide substitution model was chosen using ModelFinder Plus (MFP), and node support was evaluated with 1,000 bootstrap replicates and 1,000 SH-aLRT replicates. The six aligned enhanced DNA barcode datasets are available at https://doi.org/10.57760/sciencedb.31238.

## Limitations

None.

## Ethics Statement

All authors have read and followed the ethical requirements for publication in *Data in Brief* and confirm that the current work does not involve human subjects, animal experiments, or any data collected from social media platforms.

## CRediT Author Statement

**Xiaoman Wang:** Conceptualization, Methodology, Original draft preparation; **Kai Mu:** Software, Visualization, Writing–review & editing; **Chao Xu:** Conceptualization, Data curation, Writing–review & editing; **Zhengge Zhu:** Supervision, Project administration, Writing–review & editing; **Xueying Yang:** Funding, Supervision, Validation; **Jin Zhang:** Funding, Project administration, Writing- Reviewing and Editing.

## Data Availability

Science Data BankEnhanced DNA barcode datasets for Aristida grasses: new whole genome skimming data for the protected species Aristida triseta Keng integrated with public sequences. (Original data)
NCBI BiosampleThe BioSample accession is SAMN56514143. (Original data)
GenBankThe chloroplast genome sequence was deposited in GenBank under accession no. PZ161599. (Original data)
INSDC repositoriesThe raw sequencing reads were deposited in the SRA under accession no. SRR37669847. (Original data)
INSDC repositoriesThe BioProject accession is PRJNA1437527. (Original data)

## Appendix Supplementary materials

Image, application 1
